# Irisin as a Novel Biomarker of Subclinical Atherosclerosis, Cardiovascular Risk and Severe Disease in Axial Spondyloarthritis

**DOI:** 10.3389/fimmu.2022.894171

**Published:** 2022-07-08

**Authors:** Sara Remuzgo-Martínez, Javier Rueda-Gotor, Verónica Pulito-Cueto, Raquel López-Mejías, Alfonso Corrales, Leticia Lera-Gómez, Raquel Pérez-Fernández, Virginia Portilla, Íñigo González-Mazón, Ricardo Blanco, Rosa Expósito, Cristina Mata, Javier Llorca, Vanesa Hernández-Hernández, Carlos Rodríguez-Lozano, Nuria Barbarroja, Rafaela Ortega-Castro, Esther Vicente, Cristina Fernández-Carballido, María Paz Martínez-Vidal, David Castro-Corredor, Joaquín Anino-Fernández, Diana Peiteado, Chamaida Plasencia-Rodríguez, Eva Galíndez-Agirregoikoa, María Luz García-Vivar, Nuria Vegas-Revenga, Irati Urionaguena, Oreste Gualillo, Juan Carlos Quevedo-Abeledo, Santos Castañeda, Iván Ferraz-Amaro, Miguel Á. González-Gay, Fernanda Genre

**Affiliations:** ^1^ Research group on genetic epidemiology and atherosclerosis in systemic diseases and in metabolic diseases of the musculoskeletal system, Instituto de Investigación Sanitaria IDIVAL, Hospital Universitario Marqués de Valdecilla, Santander, Spain; ^2^ Rheumatology Division, Hospital Comarcal de Laredo, Laredo, Spain; ^3^ Department of Epidemiology and Computational Biology, School of Medicine, Universidad de Cantabria, Santander, Spain; ^4^ Consorcio Centro de Investigación Biomédica en Red de Epidemiología y Salud Pública (CIBERESP), Santander, Spain; ^5^ Rheumatology Division, Hospital Universitario de Canarias, Santa Cruz de Tenerife, Spain; ^6^ Rheumatology Division, Hospital Universitario de Gran Canaria Dr. Negŕın, Las Palmas de Gran Canaria, Spain; ^7^ Rheumatology Division, Hospital Reina Sofía, Maimonides Institute for Research in Biomedicine of Cordoba (IMIBIC), Universidad de Córdoba, Córdoba, Spain; ^8^ Rheumatology Division, Hospital Universitario de La Princesa, IIS-Princesa, Madrid, Spain; ^9^ Rheumatology Division, Hospital Universitario de San Juan, Alicante, Spain; ^10^ Rheumatology Division, Hospital General Universitario de Alicante, Alicante, Spain; ^11^ Rheumatology Division, Hospital General Universitario de Ciudad Real, Ciudad Real, Spain; ^12^ Rheumatology Division, Hospital Universitario La Paz-IdiPaz, Madrid, Spain; ^13^ Rheumatology Division, Hospital Universitario Basurto, Bilbao, Spain; ^14^ Rheumatology Division, Hospital Galdakao-Usansolo, Galdakao, Spain; ^15^ Servicio Gallego de Salud (SERGAS) and Instituto para el Desarrollo e Integración de la Sanidad (IDIS), Neuroendocrine Interactions in Rheumatic and Inflammatory Diseases (NEIRID) Lab, Research Laboratory 9, Hospital Cl´ınico Universitario de Santiago, Santiago de Compostela, Spain; ^16^ Medicine and Psychiatry Department, Universidad de Cantabria, Santander, Spain; ^17^ Rheumatology Division, Hospital Universitario Marqués de Valdecilla, Santander, Spain; ^18^ Cardiovascular Pathophysiology and Genomics Research Unit, School of Physiology, Faculty of Health Sciences, University of the Witwatersrand, Johannesburg, South Africa

**Keywords:** irisin, axial spondyloarthritis, biomarker, subclinical atherosclerosis, cardiovascular risk, disease severity

## Abstract

**Introduction:**

Patients with axial spondyloarthritis (axSpA) have a high disease burden mainly due to the rheumatic disease itself, and also exhibit accelerated atherosclerosis, that leads to a higher incidence of cardiovascular (CV) disease. Accordingly, the identification of biomarkers of CV risk and inflammation in axSpA patients is clinically relevant. In this sense, given the beneficial functions exerted by the adipomyokine irisin in processes related to CV disease and inflammation, our aim was to assess, for the first time, the role of irisin as a genetic and serological biomarker of subclinical atherosclerosis, CV risk and disease severity in axSpA patients.

**Methods:**

A large cohort of 725 Spanish patients with axSpA was included. Subclinical atherosclerosis (presence of plaques and abnormal carotid intima-media thickness values) was evaluated by carotid ultrasound. Four *irisin* polymorphisms (rs16835198 G/T, rs3480 A/G, rs726344 G/A, and rs1570569 G/T) were genotyped by TaqMan probes. Additionally, serum irisin levels were determined by ELISA.

**Results:**

Low irisin levels were linked to the presence of plaques (p=0.002) and atherogenic index values ≥4 (p=0.01). Serum irisin were positively correlated with C-peptide levels (p<0.001) and negatively correlated with visual analogue scale and Bath Ankylosing Spondylitis Metrology Index (p<0.05 in all the cases). Moreover, lower irisin levels were observed in patients with sacroiliitis and in those with a negative HLA-B27 status (p<0.001 and p=0.006, respectively), as well as in those treated with non-steroidal anti-inflammatory drugs and conventional disease-modifying antirheumatic drugs (p<0.001 and p=0.002, respectively). Interestingly, the TT genotype and the T allele of rs16835198 were less frequent in axSpA patients with ASDAS >2.1 (Odds Ratio (OR): 0.48 [0.28-0.83] and OR: 0.73 [0.57-0.92], respectively, p=0.01 in both cases). Additionally, the frequency of rs1570569 T allele was higher in these patients (OR: 1.46 [1.08-1.97], p=0.01). Furthermore, the GGGT haplotype was more frequent in patients with ASDAS values >2.1 (OR: 1.73 [1.13-2.66], p=0.01).

**Conclusions:**

Our results indicate that low serum irisin levels could be indicators of the presence of subclinical atherosclerosis, high CV risk and more severe disease in axSpA patients. In addition, *irisin* may also constitute a genetic biomarker of disease activity in axSpA.

## Introduction

Axial spondyloarthritis (axSpA) is a chronic inflammatory disease that mainly affects the axial skeleton (spine and pelvic joints), although its main symptoms can also be accompanied by extra-articular manifestations (1). axSpA has detrimental effects on the health status of the patients affected by this condition including, but not limited to, pain, stiffness and poor physical function (2). Additionally, the higher prevalence of traditional risk factors and the systemic inflammatory state of these patients contributes to an increased cardiovascular (CV) risk (3–5), being CV disease one of the leading causes of death in axSpA. In most of the cases, this high CV risk is reflected by a process of accelerated atherosclerosis, which can be assessed at the subclinical level by carotid ultrasound (US), a non-invasive imaging technique (6). By this means, the existence of surrogate markers of subclinical atherosclerosis such as abnormal carotid intima-media thickness (cIMT) values or the presence of carotid plaques can be determined (6–8).

Importantly, abnormalities in a growing number of molecules mainly implicated in metabolic and inflammatory mechanisms also boost the atherosclerotic process, further promoting the increased CV morbidity in axSpA patients (9). In this regard, muscle and adipose tissue play a pivotal homeostatic function by producing a large number of these molecules, mainly myokines and adipokines, which exert autocrine, paracrine and/or endocrine effects, affecting multiple organs (10–12). Thereby, these molecules are implicated in the regulation of the immune response and in the pathogenesis of numerous chronic inflammatory diseases (13). In this context, since its discovery in 2012, much attention has been paid to the adipomyokine irisin (14). This molecule has been reported to play a critical beneficial role in several processes such as inflammation, angiogenesis, oxidative stress, endothelial cell dysfunction, and lipid and bone metabolism (11–19). In particular, it has been described that irisin plays key roles against vascular inflammation by inhibiting the recruitment of inflammatory cells to the atherosclerotic lesions and also inducing the switch from the pro-inflammatory (M1) phenotype of macrophages to the anti-inflammatory (M2) phenotype (19–23). In addition, previous studies reported that irisin downregulates other pro-inflammatory pathways, suppressing thereby the secretion of pro-inflammatory cytokines (24). Consequently, the potential of irisin as a biomarker promoted multiple research in diverse pathological conditions, including CV-related diseases, autoimmune and chronic inflammatory diseases, osteoporosis, and different types of cancer (12, 19). Interestingly, the levels of circulating irisin seem to be influenced by the pathological status of each disease (12, 25).

Therefore, based on the above, it seems plausible that irisin could be a key molecule in axSpA since most of the processes it influences are disrupted in this condition. Surprisingly, to the best of our knowledge, there are no previous studies on the implication of irisin in atherosclerotic disease and CV risk in the context of this rheumatic disorder. In like manner, information about the potential role of irisin in the pathogenesis of axSpA is scarce.

Taking all this into consideration, in this study we aimed to evaluate for the first time the role of irisin as a genetic and serological biomarker of subclinical atherosclerosis and CV risk in a large cohort of Caucasian patients with axSpA. Furthermore, we also assessed its role as a potential marker of axSpA severity.

## Material and Methods

### Patients

A total of 725 Spanish patients who fulfilled the Assessment of SpondyloArthritis international Society classification criteria for axSpA (26) were included in this study. All these patients belong to the *AtheSpAin* cohort, a Spanish multicenter cohort to study atherosclerosis in axSpA, and were recruited at the following centers: Hospital Universitario Marqués de Valdecilla (Santander), Hospital Comarcal de Laredo (Laredo), Hospital Universitario de Canarias (Santa Cruz de Tenerife), Hospital Universitario de Gran Canaria Dr. Negrín (Las Palmas de Gran Canaria), Hospital Universitario Reina Sofía (Córdoba), Hospital Universitario de La Princesa (Madrid), Hospital General Universitario de Elda (Elda), Hospital General Universitario de Ciudad Real (Ciudad Real), Hospital Universitario La Paz (Madrid), Hospital Universitario Basurto (Bilbao) and Hospital Universitario de Galdakao (Galdakao). Patients with diabetes mellitus or chronic kidney disease were excluded from this study.

Peripheral blood samples were collected in the fasting state from all the patients at the time of recruitment. In addition, data on sex, age, body mass index, blood pressure, total cholesterol, high-density lipoprotein-cholesterol, low-density lipoprotein-cholesterol, triglycerides, C-peptide, C-reactive protein (CRP) and erythrocyte sedimentation rate (ESR) at the time of study, as well as history of traditional CV risk factors (smoking, obesity, dyslipidemia and hypertension) were collected. Obesity, dyslipidemia, and hypertension were defined as previously described (27). In particular, the atherogenic index (AI) was calculated as total cholesterol divided by high-density lipoprotein-cholesterol values. AI values ≥4 were considered as indicative of adverse lipid profile. Furthermore, clinical characteristics of the patients were also retrieved from medical records. In this regard, the clinical index of disease activity Ankylosing Spondylitis Disease Activity Score (ASDAS) was assessed, being values >2.1 considered as indicative of high disease activity. The main demographic, clinical and CV disease-related characteristics of patients as well as the treatments received (non-steroidal anti-inflammatory drugs (NSAIDs), conventional and biologic disease-modifying antirheumatic drugs (DMARDs), and statins) are displayed in **Table 1**.

**Table 1:** Demographic, clinical and cardiovascular disease-related characteristics in patients with axSpA.

Variable	axSpA
Men/Women, n	490/235
Age (years), median [IQR]	47.0 [39.0-57.0]
Age at axSpA diagnosis (years), median [IQR]	36.0 [28.0-44.0]
CRP (mg/L), median [IQR]	2.2 [0.6-6.2]
ESR (mm/1st hour), median [IQR]	6.0 [3.0-13.0]
VAS patient, median [IQR]	4.0 [2.0-6.0]
VAS physician, median [IQR]	3.0 [1.0-5.0]
BASMI, median [IQR]	2.2 [1.0-3.8]
ASDAS, median [IQR]	2.2 [1.5-3.0]
ASDAS >2.1, % (n/N)	53.4 (340/637)
HLA-B27 positive status, % (n/N)	74.0 (513/693)
Syndesmophytes, % (n/N)	40.1 (272/678)
History of peripheral synovitis, % (n/N)	35.1 (254/723)
History of enthesitis, % (n/N)	30.6 (221/722)
History of sacroiliitis^1^, % (n/N)	71.5 (266/372)
Extra-articular manifestations^2^, % (n/N)	34.6 (250/723)
History of classic cardiovascular risk factors, % (n/N)	
Smoking	53.1 (382/719)
Obesity	20.8 (150/720)
Dyslipidemia	30.8 (222/722)
Hypertension	23.8 (172/722)
Body mass index (kg/m^2^), median [IQR]	26.2 [23.7-29.4]
Systolic blood pressure (mm Hg), median [IQR]	129.0 [116.0-140.0]
Diastolic blood pressure (mm Hg), median [IQR]	79.0 [71.0-86.0]
Total cholesterol (mg/dL), median [IQR]	189.0 [165.0-214.0]
High-density lipoprotein-cholesterol (mg/dL), median [IQR]	52.0 [44.0-63.0]
Low-density lipoprotein-cholesterol (mg/dL), median [IQR]	115.0 [94.0-137.8]
Triglycerides (mg/dL), median [IQR]	96.0 [70.0-137.0]
Atherogenic index ≥4, % (n/N)	36.8 (252/684)
C-peptide (ng/mL), median [IQR]	1.5 [0.8-2.6]
Carotid IMT (mm), median [IQR]	0.618 [0.544-0.718]
Carotid plaques, % (n/N)	30.8 (223/725)
Treatment, % (n/N)	
NSAIDs	83.1 (599/721)
Conventional DMARDs^3^	36.1 (261/723)
Biologic DMARDs	38.9 (268/689)
Anti-TNF-α	94.4 (253/268)
Anti-IL17	5.6 (15/268)
Statins	14.9 (98/656)

ASDAS, Ankylosing Spondylitis Disease Activity Score; axSpA, Axial spondyloarthritis; BASMI, Bath Ankylosing Spondylitis Metrology Index; CRP, C-reactive protein; DMARDs, disease-modifying antirheumatic drugs; ESR, Erythrocyte sedimentation rate; HLA, Human leukocyte antigen; IMT, Intima-Media Thickness; IQR, Interquartile range; NSAIDs, non-steroidal anti-inflammatory drugs; SD, Standard Deviation; VAS, Visual Analogue Scale.

^1^Detected by magnetic resonance imaging. ^2^Including anterior uveitis, psoriasis and/or inflammatory bowel disease. ^3^Including methotrexate, leflunomide and sulfasalazine.

All the individuals gave their informed written consent to be included in the study. All the experiments involving humans and human blood samples were carried out in accordance with the approved guidelines and regulations, according to the Declaration of Helsinki.

### Carotid US Study

The presence of abnormal cIMT values in the common carotid artery and the presence of focal plaques in the extracranial carotid tree were assessed by carotid US in all the axSpA patients, as previously reported (6).

### *Irisin* Polymorphisms Selection and Genotyping

Deoxyribonucleic acid of patients was obtained from peripheral blood using standard procedures. All the individuals were genotyped for *irisin* rs16835198 (G/T), rs3480 (A/G), rs726344 (G/A) and rs1570569 (G/T), previously linked with CV risk factors (28–33), using pre-designed TaqMan probes (C:34204885_10, C:_8822841_10, C::927694_10 and C:_8854681_10, respectively). Genotyping was performed in a QuantStudio™ 7 Flex Real-Time polymerase chain reaction system, according to the conditions recommended by the manufacturer (Applied Biosystems, Foster City, CA, USA). Negative controls and duplicate samples were included to check the accuracy of the genotyping.

### Assessment of Irisin Serum Levels

Serum irisin levels were determined by a commercial Enzyme-Linked ImmunoSorbent Assay kit in all the axSpA patients (RAG018R, BioVendor, Brno, Czech Republic), according to the manufacturer’s instructions. All the samples were analyzed in duplicate and quantified relative to a standard curve, using 4-parameter logistic regression through MyAssays^®^ online software.

### Statistical Analysis

Shapiro-Wilk test was used to determine whether the different variables included in this study followed or not normal distribution. Data are expressed as mean ± standard deviation (SD), median [interquartile range (IQR)], number of individuals (n) or percentage (%), depending on the type of data. Serum levels of irisin were log transformed and are expressed as log_(serum irisin)_.

The relationship between genotypes, alleles, or haplotypes and categorical variables was tested using logistic regression, adjusting for potential confounding factors (sex, age at the time of the study, and classic CV risk factors). Strength of associations were estimated using odds ratios (OR) and 95% confidence intervals (CI). The association of genotypes, alleles, or haplotypes with continuous variables was evaluated by linear regression, adjusting for the potential confounding factors above mentioned. In both cases, the most frequent genotype and allele of *irisin* rs16835198, rs3480, rs726344 and rs1570569, as well as the haplotype with the highest frequency, were used as reference.

The association of serum levels of irisin with categorical and continuous variables was assessed by linear regression and Pearson’s partial correlation coefficient (r), respectively. In all the cases, adjustment was performed for potential confounding factors: sex, age at the time of the study, and classic CV risk factors.

Statistical significance was defined as *p* values ≤0.05 (or ≤0.01 in the genetic analyses applying Bonferroni correction for multiple comparisons). All the analyses were performed using STATA^®^ v.11.1 statistical software (Stata Corp, College Station, TX, USA).

## Results

### Association Between Irisin and Surrogate Markers of Subclinical Atherosclerosis and CV Disease-Related Features

**Figure 1:**
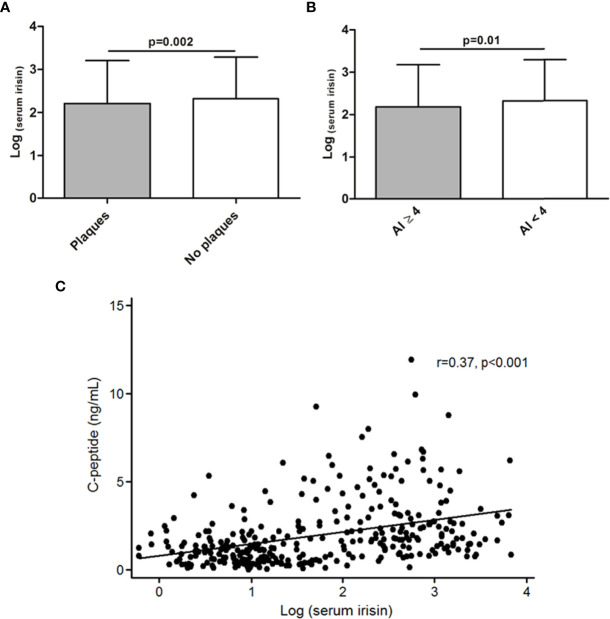
Association of serum irisin levels and features related to subclinical atherosclerosis and cardiovascular disease-related features in axSpA. Lower levels of irisin in patients with carotid plaques **(A)** and in patients with an atherogenic index (AI) ≥4 **(B)**. Positive correlation between serum irisin and C-peptide levels **(C)**. Results shown in **(A)** and **(B)** were obtained after linear regression analysis, while results shown in **(C)** were obtained after Pearson’s partial correlation test, in all the cases adjusting for sex, age at the time of the study and classical cardiovascular risk factors.

The levels of serum irisin were lower in patients who presented carotid plaques compared to those without plaques (2.21 ± 0.99 *vs* 2.32 ± 0.96, respectively, p=0.002, **Figure 1A**). Furthermore, axSpA patients with an AI ≥4 exhibited lower serum irisin levels when compared to those with an AI<4 (2.19 ± 0.99 *vs* 2.33 ± 0.97, p=0.01, respectively, **Figure 1B**). In addition, we also noted a positive correlation between serum irisin and C-peptide levels (r=0.37, p<0.001, **Figure 1C**).

No relationship was found between serum irisin and other CV disease-related characteristics. Similarly, no association was disclosed between *irisin* rs16835198, rs3480, rs726344 or rs1570569 and surrogate markers of subclinical atherosclerosis when assessing these polymorphisms individually at the genotype or allele level or when combined conforming haplotypes.

### Relationship of Irisin With Markers of Inflammation, Disease Activity, Other axSpA Features and Treatments Received

**Figure 2:**
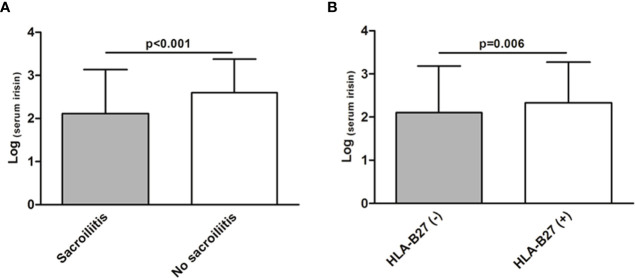
Association between serum irisin levels and axSpA-related features. Lower serum levels of irisin in patients with sacroiliitis **(A)** and in patients with a negative human leukocyte antigen (HLA)-B27 status **(B)**. Results shown in **(A)** and **(B)** were obtained after linear regression analysis, adjusting for sex, age at the time of the study and classical cardiovascular risk factors.

We observed a negative correlation of serum irisin levels with visual analogue scale (VAS) patient, VAS physician and Bath Ankylosing Spondylitis Metrology Index (BASMI) (r=-0.12, p=0.003; r=-0.19, p<0.001; r=-0.13, p=0.002; respectively). Also in this line, patients with sacroiliitis showed lower serum levels of irisin compared to those patients without this axSpA feature (2.12 ± 1.02 in patients with sacroiliitis *vs* 2.60 ± 0.78, respectively, p<0.001, **Figure 2A**). Moreover, patients with human leukocyte antigen (HLA)-B27 negative status exhibited lower serum irisin levels than those with HLA-B27 positive status (2.10 ± 1.08 *vs* 2.33 ± 0.94, respectively, p=0.006, **Figure 2B**). Regarding the treatment, we observed that patients treated with conventional DMARDs and NSAIDs showed lower serum irisin levels than those patients who were not receiving these therapies (2.13 ± 1.02 *vs* 2.37 ± 0.94 for conventional DMARDs and 2.22 ± 1.01 *vs* 2.65 ± 0.66 for NSAIDs, p=0.002 and p<0.001, respectively). Furthermore, we disclosed that patients undergoing anti-IL17 therapy presented higher serum irisin levels than those receiving anti-TNF-α treatment (2.76 ± 0.74 *vs* 2.23 ± 0.98, respectively, p=0.05).

At the genetic level, we found that the TT genotype and the T allele of rs16835198 were less frequent in axSpA patients with ASDAS values >2.1 (9.7% *vs* 14.5%, OR: 0.48 [0.28-0.83] and 32.0% *vs* 38.0%, OR: 0.73 [0.57-0.92], respectively, p=0.01 in both cases, **Table 2**). In contrast, we found that the frequency of the minor allele of rs1570569 (T) was higher in this group of patients (20.7% *vs* 16.0%, OR: 1.46 [1.08-1.97], p=0.01, **Table 2**). Moreover, the *irisin* GGGT haplotype was more frequent in axSpA patients with ASDAS values >2.1 (11.6% *vs* 7.2%, OR: 1.73 [1.13-2.66], p=0.01, **Table 2**).

**Table 2:** Genotypes, alleles and haplotypes of *irisin* according to ASDAS values >2.1 in axSpA patients.

*Irisin*	ASDAS >2.1			
polymorphism	Yes (% (n))	No (% (n))	OR [95% CI]	p*
**rs16835198**				
GG	45.7 (155)	38.4 (114)	1 (Reference)	–
GT	44.6 (151)	47.1 (140)	0.79 [0.56-1.11]	0.17
TT	9.7 (33)	14.5 (43)	**0.48 [0.28-0.83]**	**0.01**
G	68.0 (461)	62.0 (368)	1 (Reference)	–
T	32.0 (217)	38.0 (226)	**0.73 [0.57-0.92]**	**0.01**
**rs3480**				
AA	34.1 (116)	36.2 (107)	1 (Reference)	–
AG	45.9 (156)	49.3 (146)	1.10 [0.77-1.58]	0.61
GG	20.0 (68)	14.5 (43)	1.60 [0.99-2.59]	0.06
A	57.1 (388)	60.8 (360)	1 (Reference)	–
G	42.9 (292)	39.2 (232)	1.23 [0.98-1.56]	0.08
**rs726344**				
GG	77.6 (264)	74.7 (222)	1 (Reference)	–
GA	20.3 (69)	23.9 (71)	0.80 [0.54-1.18]	0.25
AA	2.1 (7)	1.4 (4)	1.42 [0.39-5.12]	0.60
G	87.8 (597)	86.7 (515)	1 (Reference)	–
A	12.2 (83)	13.3 (79)	0.89 [0.63-1.24]	0.48
**rs1570569**				
GG	63.3 (214)	70.1 (206)	1 (Reference)	–
GT	32.0 (108)	27.9 (82)	1.38 [0.96-1.97]	0.08
TT	4.7 (16)	2.0 (6)	2.72 [1.02-7.26]	0.05
G	79.3 (536)	84.0 (494)	1 (Reference)	–
T	20.7 (140)	16.0 (94)	**1.46 [1.08-1.97]**	**0.01**
Haplotype**	**ASDAS >2.1**			
	**Yes (% (n))**	**No (% (n))**	**OR [95% CI]**	p*
GAGG	38.4 (259)	38.7 (227)	1 (Reference)	–
TAGG	18.1 (122)	22.2 (130)	0.75 [0.55-1.03]	0.07
GGGT	11.6 (78)	7.2 (42)	**1.73 [1.13-2.66]**	**0.01**
GGGG	10.5 (71)	7.5 (44)	1.47 [0.96-2.25]	0.08
TGGT	6.1 (41)	6.3 (37)	1.01 [0.62-1.66]	0.97
GGAG	4.7 (32)	5.8 (34)	0.79 [0.46-1.34]	0.38
TGAG	4.3 (29)	4.9 (29)	0.86 [0.49-1.50]	0.59

ASDAS, Ankylosing Spondylitis Disease Activity Score; axSpA, axial spondyloarthritis; SD, standard deviation.

Results obtained after logistic regression analysis. *p-values adjusted for sex, age at the time of the study and classical cardiovascular risk factors. **The polymorphism order was rs16835198, rs3480, rs726344 and rs1570569. Haplotypes with a frequency higher than 4% are shown. Statistically significant results are highlighted in **bold**.

No significant associations were observed between irisin and CRP, ESR, other disease-related features or treatment with statins.

## Discussion

axSpA patients exhibit a great disease burden product not only of the rheumatic disease itself, but also due to the higher incidence of CV disease, which currently constitutes one of the main causes of death in these patients (3, 4). Consequently, the identification of molecules implicated in the development of subclinical atherosclerosis and CV disease in axSpA that may be used as biomarkers of CV risk and inflammation in these patients is clinically relevant. Accordingly, given the important functions exerted by irisin in different processes mainly related to CV disease and inflammation, our aim was to assess for the first time the role of irisin as a biomarker of subclinical atherosclerosis, CV risk and disease severity in axSpA patients.

Regarding the potential implication of irisin as a biomarker of CV risk in axSpA, we found that low levels of serum irisin were associated to the presence of carotid plaques, an indicator of an advanced stage of atherosclerosis and high CV risk (8). These results are in accordance with studies performed in other conditions, which report that circulating irisin levels are inversely linked to the burden of coronary atherosclerosis, vascular calcification and severity of coronary artery disease (20, 34–38). These results are further supported by our finding that low serum irisin levels are associated with AI indicative of adverse lipid profiles in our patients. In this respect, other authors also reported that higher serum levels of irisin are related to more favorable lipid profiles in the general population (34, 39). Accordingly, both results are in agreement with the atheroprotective and anti-inflammatory role proposed for irisin in pathological contexts different from axSpA (19–24). In this regard, a relevant role for irisin against vascular inflammation, endothelial cell dysfunction, oxidative stress and plaque progression has been described (20–23). Interestingly, in favor of the anti-inflammatory function of irisin, we also disclosed that serum irisin positively correlated with C-peptide, another molecule with similar beneficial effects on inflammation (40). Of note, a previous study performed in patients with type 2 diabetes mellitus reported an inverse association between irisin and interleukin (IL)-17A (41), one of the main pro-inflammatory cytokines implicated in the pathogenesis of axSpA (1). IL-17A was suggested to exert an indirect pro-atherosclerotic role in obese individuals (42). Hence, these data strengthen our results on the anti-inflammatory and anti-atherogenic role of irisin in axSpA.

Additionally, we noted that low levels of serum irisin were associated with features linked to more severe disease activity in axSpA, including higher VAS scores, higher spinal mobility index BASMI and presence of sacroiliitis. Interestingly, we also found lower serum levels of irisin in axSpA patients with a negative HLA-B27 status, a subgroup of axSpA patients which has been recently reported to have higher disease activity when compared to their positive counterparts (43, 44). To the best of our knowledge, there is so far only one study that evaluated the role of irisin in ankylosing spondylitis. In such study, the authors found that patients with more severe disease symptoms exhibited lower serum levels of irisin, which is in accordance with our results (45). Also in this line, previous studies performed in other rheumatic diseases reported an inverse association between irisin serum levels and disease activity (22, 46–48). In addition, patients treated with conventional DMARDs and NSAIDs exhibited lower serum irisin levels. These results may be reflecting the worse clinical status of the patients who are receiving conventional DMARDs and NSAIDs treatment. This is in agreement with our findings that indicate an association of low serum levels of irisin with more severe disease. It is possible that biologic DMARDs may have a beneficial modulatory effect on irisin levels that may be related to clinical improvement following the use of these therapies. In particular, anti-IL17 therapy was associated with higher serum irisin levels when compared to anti-TNF-α treatment in our cohort. Nonetheless, this should be interpreted cautiously given that only 5.6% of our patients undergoing biologic therapy were receiving anti-IL17 treatment, whereas the remaining patients were being treated with anti-TNF-α.

Furthermore, our study also revealed an association between *irisin* and ASDAS values. In particular, we disclosed a protective effect of rs16835198 T allele and a risk effect for rs1570569 T allele in this regard. Moreover, the GGGT *irisin* haplotype was more frequent in patients with ASDAS values >2.1, indicative of high disease activity. To the best of our knowledge, these findings are novel since there are no previous studies in this context.

Our study has several strengths, mainly the large number of individuals with data on carotid US studies that constitute the *AtheSpAin* cohort and the fact that irisin was assessed in all of them at two molecular levels, genetic polymorphisms and protein. Nevertheless, we acknowledge that some potential limitations may exist. In this respect, in our records we do not have information on the level of physical activity of our patients, which has been described to influence on irisin serum levels (10, 19). Furthermore, regarding essential markers of inflammation, we analyzed the association of irisin with CRP and ESR, although no data was available related to other markers, such as TNF-α or IL-6.

In conclusion, our results suggest that low serum irisin levels can be indicators of the presence of subclinical atherosclerosis, high CV risk and more severe disease in axSpA patients. In addition, *irisin* may also constitute a genetic biomarker of disease activity in axSpA. Based on these results, irisin could represent a potential target of novel therapeutic strategies, aimed to prevent the development of CV disease and axSpA progression.

## Data Availability Statement

The original contributions presented in the study are included in the article/supplementary material. Further inquiries can be directed to the corresponding author.

## Ethics Statement

All experimental protocols were reviewed and approved by the Ethics Committee of research of Cantabria (for Hospital Universitario Marqués de Valdecilla, Santander, and Hospital Comarcal de Laredo, Laredo), Ethics Committee of clinical research of Complejo Hospitalario Universitario de Canarias (for Hospital Universitario de Canarias, Santa Cruz de Tenerife), Ethics Committee of clinical research of Hospital Universitario de Gran Canaria Dr. Negrín (for Hospital Universitario de Gran Canaria Dr. Negrín, Las Palmas de Gran Canaria), Ethics Committee of research of Córdoba (for Hospital Universitario Reina Sofía, Córdoba), Ethics Committee of clinical research of Madrid (for Hospital Universitario de la Princesa and Hospital Universitario La Paz, Madrid), Ethics Committee of Clinical research of Elda (for Hospital General Universitario de Elda, Elda), Ethics Committee of clinical research of Ciudad Real (for Hospital General Universitario de Ciudad Real, Ciudad Real) and Ethics Committee of research of Euskadi (for Hospital Universitario de Basurto, Bilbao and Hospital Galdakao-Usansolo, Galdakao). The patients/participants provided their written informed consent to participate in this study.

## Author Contributions

SR-M, JR-G and VP-C carried out the conception and design of the study, were involved in the statistical analysis and interpretation of data and in the drafting of the manuscript. RL-M, AC, LL-G, RP-F, VP, IG-M, RB, RE, CM, JL, VH-H, CR-L, NB, RO-C, EV, CF-C, MPM-V, DC-C, JA-F, DP, CP-R, EG-A, MLG-V, NV-R, IU, OG, JCQ-A, SC and IF-A helped in the acquisition and interpretation of data, and contributed to the elaboration of the manuscript. MAG-G and FG supervised all aspects of the research and analysis and were responsible of the final drafting and elaboration of the manuscript. All authors have approved the final article.

## Funding

This work was partially supported by grants from Instituto de Investigación Sanitaria IDIVAL (NVAL17/10) and from the ‘Asociación Cántabra de Reumatología’ awarded to FG. FG and JR-G are beneficiaries of a grant funded by ‘Instituto de Salud Carlos III’ (ISCIII) (PI20/00059). FG is supported by funds of the RICORS Program (RD21/0002/0025) from ISCIII, co-funded by the European Union. SR-M and VP-C are supported by funds of the RETICS Program (RD16/0012/0009) from ISCIII, co-funded by the European Regional Development Fund. RL-M is a recipient of a Miguel Servet type II Program fellowship from ISCIII, co-funded by the European Social Fund, `Investing in your future´ (CPII21/00004).

## Conflict of Interest

The authors declare that the research was conducted in the absence of any commercial or financial relationships that could be construed as a potential conflict of interest.

## Publisher’s Note

All claims expressed in this article are solely those of the authors and do not necessarily represent those of their affiliated organizations, or those of the publisher, the editors and the reviewers. Any product that may be evaluated in this article, or claim that may be made by its manufacturer, is not guaranteed or endorsed by the publisher.

## Abbreviations

AI, atherogenic index; ASDAS, Ankylosing Spondylitis Disease Activity Score; axSpA, axial spondyloarthritis; BASMI, Bath Ankylosing Spondylitis Metrology Index; CI, confidence interval; cIMT, carotid intima-media thickness; CRP, C-reactive protein; CV, cardiovascular; DMARDs, disease-modifying antirheumatic drugs; ESR, erythrocyte sedimentation rate; HLA, Human leukocyte antigen; IQR, interquartile range; NSAIDs, non-steroidal anti-inflammatory drugs; OR, odds ratio; SD, standard deviation; US, ultrasound; VAS, visual analogue scale.
